# Fracture of the Ribs in a Horse

**Published:** 1903-03

**Authors:** William Drinkwater

**Affiliations:** Monticello, Iowa


### FRACTURE OF THE RIBS IN A HORSE.

BY WILLIAM DRINKWATER, V.S.,
MONTICELLO, IOWA.

IN the early part of the summer of the past year, a farmer brought to my place of business a brown gelding with a large swelling on the left side of the thorax extending from about the eighth rib to the twelfth, and from about the ends of the ribs where they are extended by cartilage to the sternum to eight inches above. The history of the case was, that in the winter—which was a year previous to the past one—the horse was kicked by another. It did not seem to suffer from it for some time and did its share of the summer's work, but the past winter the owner said it got the heaves from food of a poor quality.

On the surface of the swelling were several nodules that were about ready to discharge. I made an opening in one of the more prominent ones and it discharged a quantity of serous fluid. I then explored with the fingers the various recesses of the swelling for pieces of ribs or any dead tissues, but found only blood-clot and the part bled freely, probably from the subcutaneous vein.

After clearing out the cavities, I packed them with cotton saturated with a solution of copper sulphate, zinc sulphate, and lead acetate, one ounce of each to a quart of water. My thoughts at that time were, that an astringent and caustic would stop the hemorrhage, which it did in a few minutes, and also cause some slough, and in that way reduce the enlargement. The horse was led home and brought to me again in a few days and the swelling was reduced in size. The owner said the horse had been failing and becoming useless and was not improving. I removed the cotton packing and

[1] Read before the Fifteenth Annual Meeting of the Iowa State Veterinary Medical Association, January 14 and 15, 1903.

cleansed the cavities, and filled in again with cotton and the solution. Hs was brought again in a few days, seeming a little better in spirits, but the parts affected and his breath were giving off a sickening odor. He was left in my care, and I washed the cavities daily and injected the solution and packed, but the horse would not eat much of the grass and oats offered him and weakened so, that I sent word to the owner to take him home before he died. When the owner arrived next morning the horse was dead, and we made an arrangement with a man to take the carcass out and take off the hide and let us examine the seat of the difficulty.

We found a mass of tissue being formed around an opening into the thoracic cavity where three ribs had been broken in. I removed one piece of rib three inches in length which was lying loose. The other parts were crumbled to small pieces and some pus was found in the cavity. A thickened membrane was formed to protect the lung, and the lung itself was discolored and hardened along its lower border, which I believe accounted for the heaves or broken wind condition. If there had been an operation and a thorough clearing of the lesion at a proper time, could it have resulted in relief to the patient?

